# Dietary Energy and Nutrient Intake of Healthy Pre-School Children in Hungary

**DOI:** 10.3390/nu15132989

**Published:** 2023-06-30

**Authors:** Mónika Csertő, Krisztina Mihályi, Edina Mendl, Dorottya Lőcsei, Vivien Daum, Nóra Szili, Tamás Decsi, Szimonetta Lohner

**Affiliations:** 1 Department of Paediatrics, Clinical Center, Medical School, University of Pécs, 7623 Pécs, Hungary; 2 Department of Oncotherapy, University of Pécs, 7624 Pécs, Hungary; 3 Nutrition and Dietetic Service, Directory of Nursing, Clinical Centre, University of Pécs, 7624 Pécs, Hungary; 4 Department of Public Health Medicine, Medical School, University of Pécs, 7624 Pécs, Hungary

**Keywords:** child nutrition, dietary assessment, macronutrients, micronutrients, nutrient intakes

## Abstract

Diet in the early years of life may influence the development of chronic diseases later on. The aim of the present study was to investigate the dietary intake of 2- to 6-year-old Hungarian children. In 2013 and subsequently in 2016, cross-sectional surveys were conducted among parents of healthy children attending kindergarten in Hungary. We used a three-day food diary to record quantitative data of all the nutrients consumed by the children on two working days and one weekend day. The dietary intakes were compared to both the Hungarian recommended dietary allowances and the European Food Safety Authority recommendations. The nutritional data of altogether 186 children in 2013 and 556 children in 2016 were analyzed. The total energy and carbohydrate intake was appropriate. We observed high sugar intake in every fifth child. Protein, fat and cholesterol intake, as well as the intake of sodium, potassium and phosphorus, were high. The consumption of calcium and vitamin D was low. Water consumption was not satisfying. The present results underline the need for interventions starting early in life in order to ameliorate nutrient intake during childhood, possibly impacting long-term health outcomes.

## 1. Introduction

Adequate nutrition is essential for children’s growth and development. The inadequate intake of energy and nutrients may predispose children to a wide range of childhood and adulthood health problems, such as obesity, dental caries or even poor academic achievement [1]. Non-communicable diseases (such as type-2 diabetes, hypertension or cardiovascular disease) have become worldwide leading health problems during the past few decades; it has been convincingly demonstrated that these chronic diseases might be linked to childhood diet [2,3,4,5,6]. The dietary habits acquired in childhood often persist later in life [7]; therefore, improving children’s food choices at the preschool age might be crucial to influence food choices later in life [8]. In Hungary, the dietary habits of schoolchildren aged 11 to 14 years were reported in 2007 [9]. Although the total energy intakes were appropriate, intakes of sodium were almost two- to three-fold higher than the recommended daily allowances (RDA) for these children. The ratio of *n*-6 to *n*-3 long-chain polyunsaturated fatty acids (LCPUFAs) was high, while the consumption of vitamin D was low in both genders. Another study reported the nutrition assessment of 0 to 3-year-old Hungarian infants and toddlers in four cities [10]. Hungarian children aged 0 to 3 years had high intakes of protein, sodium and phosphorus, while intakes of calcium and vitamin D were inadequately low. Moreover, the number of children with inadequately low calcium intakes increased significantly with increasing age. Intakes of iron, essential for the development of psychomotor and cognitive functions, were low in approximately 25 percent of 1- to 3-year-old children. Overweight and obesity affected 6.5 percent of the 2- to 3-year-old children, while 23.9 percent of the 24–36-month-old children were underweight.

As a response to the increasing trend in the prevalence of childhood obesity worldwide, governments initiated intervention programs to change this trend. The urgent need for new dietary, physical activity, behavioral, environmental and pharmacological approaches for the prevention and treatment of obesity, as well as for effective family-based and school-based programs, was emphasized [11,12,13]. Hungary implemented an extra tax on unhealthy food—called the “junk food tax” [14]—in 2011, mandatory physical education five times in a week for schoolchildren [15] in 2012, and the inclusion of less salt and more healthy food into the menus provided in schools in 2014 [16]. Taking into account the health and economic effects of salt reduction, the European Union developed a salt reduction framework program [17], which Hungary also joined some years ago. In our country, this program is known as Stop-Só National Salt Reduction Program [18]. The main elements of the Stop-Só National Salt Reduction Program were developed in 2009; our country undertook a 16 percent reduction in salt content in the widely consumed food groups in less than four years in order for the population to reduce salt intake. In Hungary, a kindergarten is defined as a full-time educational center for children between the ages of three and six years; however, children are allowed to be admitted from the age of 2.5 years. Most of the children receive three meals for five days a week in these daycare settings [19].

The aim of the present study was to investigate food consumption, including intakes of energy and macro- and micronutrients, in children attending a kindergarten in Hungary and compare the values observed with the current recommendations.

## 2. Materials and Methods

The data were collected in 2013 and 2016 in the baseline phase of two randomized controlled trials (2013–2014: NCT03241355 [20] and 2016–2017: NCT03457688). This way, the data correspond to a repeated cross-sectional study.

The parents of healthy children, who were attending a kindergarten in one of five Hungarian cities (Pécs, Győr, Mohács, Szeged and Szekszárd) and volunteering for one out of two consecutive prebiotic supplementation studies coordinated by our research group, were approached. The exclusion criteria were: congenital disease or malformation influencing the gastrointestinal system, immunodeficiency, food intolerance, food allergy or metabolic disorder requiring a special diet; regular (>three times per week) consumption of products or food supplements containing prebiotics or probiotics, antibiotic or laxative treatment; and/or any infectious disease within 14 days at the time of pre-examination. They were asked to fill in a three-day food-record diary and to record the quantitative data for all foods and beverages consumed by their children on two non-consecutive working days and on one weekend day (e.g., Sunday or Saturday). The three-day diary was filled in three times during the supplementation study; however, for the purpose of the cross-sectional dietary survey, we only used data from the diaries filled in at the very start of the studies. The information was collected in November to December in 2013 and in September to November in 2016.

The parents were asked not to change the eating habits of the child during the study and to record all foods and drinks consumed by the child with the precise amount of the consumed portions. In the three-day food-record diary, each day, information had to be provided on the type of foods and beverages (including water) consumed by the child on the given day (starting from the time of waking up and proceeding chronologically until the time of going to sleep). The foods were to be described in detail, including preparation methods and brands when relevant, as well as the amounts consumed.

If necessary, a qualified, skilled dietician discussed the food item data with the parents. The nursery staff was also involved into the study in order to receive proper information about food items and portion sizes consumed during the day in the kindergarten.

The energy and nutrient intake calculations were performed with NutriComp Étrend Sport 4.0 software (NutriComp Health and Nutrition Co., Budapest, Hungary) This software includes a wide range of foods available on the market and consumed in Hungary; however, also, other food items can further be added to the database. The software was validated and effectively used already in other dietary surveys in Hungary [21,22].

The average intakes over the three days were calculated to represent the observed intake distributions. The dietary data included nutrient intake estimates from foods (both naturally present and fortified) and drinks only and excluded nutrient intake estimates contributed by any dietary supplements.

The children’s height and weight were recorded with children standing barefoot in light clothing. The ratio of calculated energy intake (EI) and estimated basal metabolic rate (BMR) was used to check the subjects for misreporting. BMR was calculated according to the Hungarian recommendation [23]. The record was excluded if the EI/BMR ratio was lower than 1.1 or higher than 2.7, according to the method of Goldberg et al. [24]. The statistical analysis was performed by SPSS 20.0 (IBM). Each child’s average daily intake was compared to the Hungarian national recommendations [25] and to the recommendations of the European Food Safety Authority [26,27]. Although both recommendations are displayed, the comparisons—since they are from the data of Hungarian children—were basically compared to the Hungarian recommendation. The EFSA recommendation only provides additional information.

Seventy percent of the Hungarian RDA was defined as the lower border (potentially inadequate), whereas 130 percent of the RDA was defined as the higher border (excessive intake) to determine the extreme level of daily nutrient intake of subjects according to the method of the Institute of Medicine in the United States of America [28].

The descriptive statistics were generated and presented in order to show the mean intakes of nutrients and the proportion with adequate intakes. The results in the tables were expressed as mean, standard deviation (SD) and percentage values of the appropriate variables [29].

Two age groups were formed according to the categories of the Hungarian national nutrient recommendations: 2.5- to 4-year old children (including all children prior to four years of age at the time of the examination) and children aged 4 to 6 years (i.e., over four years but prior to seven years of age).

The prebiotic supplementation trials where these cross-sectional data were obtained were approved by the Scientific and Research Ethics Committee of the Medical Research Council, Budapest, Hungary (STUDY I: 40564-3/2013/EKU and STUDY II: 34458-1/2016/EKU). Children were included in the study after their parents have provided written informed consent.

## 3. Results

In 2013, from a total of 219 applicants, 209 children started the study and filled in the three-day food-record diary. Then, 16 children (eight percent) were excluded as possible under-reporters and 7 children (three percent) as possible over-reporters. Finally, 186 (85 percent) children had valid, reliable data suitable for statistical analysis. The final sample (*n* = 186) consisted of 51 children aged 2.5 to 4 years and 135 children aged 4 to 6 years. The gender distribution was 91 girls to 95 boys.

In 2016, out of 942 applicants, 782 children started the study. Then, 19 children (two percent) were excluded as possible under-reporters and 17 (two percent) children as over-reporters. Finally, 556 participants (300 boys and 256 girls) had valid, reliable results suitable for statistical analysis. Of these children, 148 children were 2.5 to 4 years old, and 408 children were 4 to 6 years old at the beginning of the study. All the children attended kindergarten five days per week in both studies.

### 3.1. Energy and Macronutrient Intakes

The mean daily energy and nutrient intake of children is shown in detail in Table 1 and Table 2. The percentages of children with excessive, adequate or insufficient nutrient intakes are shown in detail in Table 3 and Table 4.

In 2013, the total mean daily energy intakes in 2.5- to 4-year-old children were fully appropriate when compared to the Hungarian recommended values adjusted for age. Three years later, in 2016, more than one fourth of the 2.5- to 4-year-old children had excessive daily energy intakes. Among the 4- to 6-year-old children, in 2016, approximately three times more children had high daily energy intakes than in 2013, according to the Hungarian recommendations.

In both the age groups and in both the years investigated (2013 and 2016), the mean protein intakes were higher than the Hungarian recommended values. However, the high consumption of protein decreased with increasing age: in 2016, among the 2.5- to 4-year-old children, every second child and one quarter of the 4- to 6-year-old children had a high protein intake, respectively.

The fat consumption as a percentage of energy (E%) was between approximately 30–35 for both the age groups in both studies.

The cholesterol intakes were far above the Hungarian recommended values: 45 percent of the 2.5- to 4-year-old children had a high cholesterol intake in 2013 and 69 percent in 2016. In 2013, a total of 27 percent, and in 2016, a total of 47 percent of the 4- to 6-year-old children had a high cholesterol intake, respectively.

Among the 2.5- to 4-year-old children, the mean daily fluid intakes were 22 percent lower than the recommendations; moreover, 44 percent of the 2.5- to 4-year-old children (2016) had insufficient daily fluid intakes (according to both Hungarian and EFSA recommendations.) While the Hungarian and EFSA recommendations on daily fluid intakes for 4- to 6-year-old children are obviously higher (1600 mL/day) than those for 2.5- to 4-year-old children (1300 mL/day), the mean intakes were almost exactly the same low in this age group (in 2013: 59 percent, in 2016: 68 percent).

In 2013, the sugar consumption was high by 27 percent, and in 2016, by 39 percent of the 2.5- to 4-year-old children. The result was not much better at the age of 4 to 6 years. In 2013, a total of 40 percent, and 23 percent of the children in 2016, had a high sugar intake (the Hungarian and EFSA recommendations for sugar intake are the same).

### 3.2. Macroelements

All preschool children, without exception, had higher daily sodium intakes than the Hungarian and the EFSA recommendations. The sodium intakes in 2.5- to 4-year-old children in 2013 were almost five times higher than the corresponding Hungarian recommended daily sodium intakes (Table 1); in 2016, the corresponding values were more than six times higher than the recommendation (500 mg/day). In 4- to 6-year-old children in 2013, the mean daily sodium intakes were 3.5 times higher than the recommended daily intakes (700 mg/day), whereas in 2016, the corresponding values were approximately five times higher than the Hungarian recommendations.

Over nine out of ten of the 2.5- to 4-year-old children achieved the recommended daily potassium intakes in both studies according to both recommendations (Hungarian and EFSA). In the 4- to 6-year-old age group, the mean daily potassium intakes in 2013 were higher than the recommendations in more than half of the children, whereas in 2016, the corresponding value was higher. In contrast, the daily calcium intakes were far below the Hungarian recommendation of 800 mg/day (for both the age groups) in both of the studies (Table 1). In 2016, in the 2.5- to 4-year-old children, the mean daily calcium intakes were low for 46 percent of them, whereas in the 4- to 6-year-old children, the corresponding value was 39 percent. The intakes of phosphorus were above the recommended value (EFSA and HRDA), and with increasing age, a slight increase in those exceeding the recommended daily intake was observed. In 2016, the mean daily intakes of magnesium were higher than the recommendation by 82 percent in the 2.5- to 4-year-old children and 48 percent in the 4- to 6-year-old children.

### 3.3. Microelements

In 2016, the intakes of iron in over three quarters of the children in both age groups achieved the national recommendations. In 2016, the mean daily copper intakes of 76 percent of the 2.5- to 4-year-old children were higher than the Hungarian recommended intakes (0.4 mg/day). In over 29 percent of 4- to 6-year-old children, the intakes of copper were higher than the HRDA (0.6 mg/day). The results for 2013 were very similar to those seen in 2016. The mean daily zinc intake values were satisfactory at above 67 percent in each group in both studies compared to the Hungarian recommendations.

### 3.4. Fat Soluble Vitamins

The recommendation for vitamin A (retinol) is formulated as the retinol equivalent (recently, the retinol activity unit): 1 RAU is equal to 1 μg retinol or 12 μg β-carotene. In 2016, the mean daily intakes for both 2.5- to 4-year-old and 4- to 6-year-old children met the recommendations; however, the individual daily intakes were low in 2016 for approximately 21 percent and were high for approximately 23 percent of the 2.5- to 4-year-old children (Table 3) compared to the Hungarian recommendations. The corresponding values were 32 percent (low) and 16 percent (high) in the 4- to 6-year-old children (Table 4). In 2013, we observed similar results. The mean daily intakes of calciferols were low for nearly the entity of the 2.5- to 4-year-old children and 100% of the 4–6-year-old children according to the Hungarian recommendations (Table 3 and Table 4).

In 2016, higher than the Hungarian recommended intakes of vitamin E (α-tocopherol) were observed in 58 percent of the 2.5- to 4-year-old children. In 2013, approximately 78 percent of the 2.5- to 4-year-old children and 56 percent of the 4- to 6-year-old children had high intakes of vitamin E (Table 3 and Table 4).

### 3.5. Water Soluble Vitamins

While the mean intakes of the water soluble vitamins thiamine, riboflavin, pyridoxine, and cobalamin considerably exceeded the recommendation, the ascorbic acid intakes were around the recommended levels in each age group in both studies according to both recommendations (Table 1 and Table 2).

## 4. Discussion

Our study showed that in Hungarian preschool children, sugar, protein, fat, cholesterol, sodium, potassium and phosphorus intakes were, in general, high, whereas intakes of calcium, vitamin D, pantothenic acid and folic acid were low. Water consumption was not satisfying in either of the age groups. Moreover, among the 2.5- to 4-year-old children, every third child had an excessive intake of energy according to the recommendations of either HRDA or EFSA.

The importance of dietary intervention programs for preschool children to influence theirs food choice at an early age in order to prevent several childhood and adulthood health problems is evident. In Hungary, kindergartens provide pre-school education and full day care for children aged 2.5 to 6 years as part of the public education system. Kindergarten education and care is free in all public institutions; moreover, at the time of this survey, approximately one third of the Hungarian 3- to 6-year-old children received meals in kindergarten for free (parents only have to pay for the meals if their income is above a certain level). Children consumed not only one hot meal (lunch) in the childcare institution but also two other cold meals (one in the morning hours and one in the afternoon). This means that the foods and beverages consumed in the kindergarten are core parts of the children’s diets.

All the kindergartens included into the present study were maintained within the Hungarian public education system, i.e., privately-owned kindergartens offering various special services for an extra fee were not included. Since in public kindergartens, both the admission criteria of the children and the fees to be covered by the families are regulated on a nation-wide basis, it can be assumed with good reason that the socio-economical backgrounds of the families in the present study were evenly distributed and representative for the 2.5- to 6-year-old urban pediatric population in Hungary.

The energy intake data seen in our dietary survey indicate that a positive energy balance is already present in a considerable percentage of kindergarten-aged children in Hungary. Worldwide, over 200 million children (one in three children under five) are either undernourished or overweight. The proportion of overweight children (5 to 19 years old) rose from one in ten to almost one in five in roughly one generation’s time between 2000 and 2016 [30]. This also underlines the importance of the early start of dietary intervention programs to prevent obesity. In Hungary, the public kindergarten system may offer an excellent place for carrying out obesity prevention programs.

Adequate protein and essential amino acid intakes are important for normal child growth and development; however, a high protein intake has no known benefit but carries an additional possible risk of obesity development. The background of this phenomenon might be explained by the “Early Protein Hypothesis”: excessive intakes of protein stimulates the secretion of insulin and insulin-like growth factor I (IGF-1) and increases the plasma concentration of insulin-releasing amino acids [31]. The high protein intakes observed in the present study may contribute to susceptibility for overweight and obesity.

Long periods of adequate Ca intake in childhood increase bone mineral density (BMD) and reduce osteopenia risk [32]. Both in the present Hungarian dietary questionnaire study and in a similar study among 6-year-old Polish children, intakes of calcium did not reach the recommended level, and inadequate intakes of vitamin D and potassium with excessive intakes of sodium were observed [33]. The observation that a dietary pattern characterized by a relatively high consumption of dairy products and whole grains with cheese and eggs is positively associated with childhood higher bone mineral density [34] might offer the possibility of dietary intervention.

Studies indicated that the early introduction to starchy table foods resulted in an increased affinity for the taste of salt at the preschool age [35]. Sugar-sweetened beverage consumption was described to be positively associated with salt intake; each additional 1 g/day salt intake was associated with 17 g/day sugar-sweetened beverage consumption in a study [36]. Consequently, reducing the salt intake might have a preventive role in both later hypertension and childhood obesity.

Although in our investigation, the energy percentage derived from protein, fat and carbohydrates were in their normal ranges, the energy percentage of added sugar was one to two percent higher than recommended. A high intake of sugar-added beverages is associated with an increased risk of overweight and obesity [37,38,39]. Furthermore, it might result in higher diastolic blood pressure and elevated triglycerides levels and elevated cardiovascular risks later in life [40].

Excessive salt intake causes extracellular volume increase, resulting in an elevation of blood pressure [41].

The uppermost recommended daily intake of sodium is 0.5 g for 1- to 3-year-old children and 0.7 g for 4- to 6-year-old children (according to HRDA). The current intakes of sodium in the present study were three to five times higher than the Hungarian recommended daily sodium intake for preschool children. The Public Catering Act-EMMI (Ministry of Human Capacities) Decree 37/2014. (IV.30) [16] maximized the quantity of added salt and decreased it to 2.5 times lower than before. This regulation (“Canteen reform”) has been mandatory for kindergartens, primary and secondary schools, in-patient care and any other public catering in Hungary since September 2015. During implementation, it is important to keep in mind that most of the sodium originates from processed foodstuffs, not mainly from table salting.

Excessive refined sugar and sodium intake and suboptimal calcium were described not only in other Hungarian cross-sectional surveys involving children from other age groups [9,10] but also in other countries [32,33] involving children from the age groups investigated in our study. This also underlines the extent of the problem and strengthens the importance of the early healthy-eating interventions among the kindergarten and preschool children.

Our present study has some limitations. Some of the data originated from the kindergarten teachers, who completed the filling out of the questionnaires in addition to their other work duties, which could have resulted in inaccuracies in data intake and later during the evaluation. The dietary data included nutrient intake estimates from foods (both naturally present and fortified) and drinks only and excluded nutrient intake estimates contributed by eventual dietary supplements. The differences in the results of the two studies may be due to the larger sample size in 2016.

Children acquire dietary habits during early life. It should be, therefore, a high priority to provide evidence-based and parent-focused practical support for parents and emphasize the importance of healthy eating among the staff members of kindergarten kitchens [42]. Inappropriate diets and eating habits in childhood lead to an increasing number of overweight and obese children and adolescents, although there is an increasing awareness of the adverse effects of non-communicable diseases. In Hungary, the obesity rate is high; among children, it is 40 percent, and among adolescents, it is 32 percent [43]. Family and public catering systems (e.g., preschool, school) have a predominant place in the formation of nutritional behavior. There are several risk behaviors, e.g., elevated energy and fat intake, the overconsumption of simple carbohydrates, excess salt intake, low vegetable and fruit consumption, low calcium intake and the consumption of sugar-sweetened beverages among kindergarten and school children. The objective is reducing the prevalence of obesity and non-communicable diseases, making the healthier option the easier option.

## 5. Conclusions

Sodium and refined sugar intake, which are known risk factors of obesity and high-blood-pressure disease, should be controlled already in kindergarten children. An increased consumption of dairy products should be advised to increase the calcium intake. The average daily intake of vitamin D was insufficient for every child. Pantothenic acid and folic acid intake were low. Water and dietary fiber consumption was not satisfying.

While the total energy and carbohydrate intake was appropriate among the younger observed group, every third child had an excessive intake of energy. Protein, fat and cholesterol intake was, in general, high.

Sodium intake was enormously high, while potassium intake also exceeded the recommended value in both studies. It results in a rather unfavorable sodium/potassium ratio. Interventions should be focused on the promotion of fruit, vegetable and water consumption. Furthermore, a decrease in saturated fat and added sugar intake is needed.

## Figures and Tables

**Table 1:** Dietary energy and nutrient intake of healthy, 2.5- to 4-year-old pre-school children in Hungary.

Daily Energy/Nutrient Intake	Survey in 2013 2.5–4 Years (*n* = 51) Mean Age: 3.3 Years Min Age: 2.6 Years Max Age: 3.9 Years		Survey in 2016 2.5–4 Years (*n* = 148) Mean Age: 3.4 Years Min Age: 2.9 Years Max Age: 3.9 Years		Hungarian Recommendation 1–3 Years	EFSA Recommendation * 1–3 Years
	Mean	SD	Mean	SD		
Energy (kcal)	**1435**	237	1579	263	1350	4.75 MJ/day, at PAL = 1.4
Protein (g)	52	10	58	12	43	0.9 g/kg bw/d
Protein (E%)	15	3	15	3	13	-
Fat (g)	49	12	58	13	44	-
Fat (E%)	32	8	34	8	30	35–40
Carbohydrate (g)	194	35	202	40	188	121–161
Carbohydrate (E%)	55	10	52	10	57	45–60
Cholesterol (mg)	177	70	212	64	135	no data
Dietary fiber (g)	19	28	16	5	15	10
Water (mL)	1088	287	1034	719	1300	1300
Sugar (E%)	11	5	11	4	10	10
Sodium (mg)	2355	703	3162	767	500	400
Potassium (mg)	1979	469	1946	436	1000	800
Calcium (mg)	627	256	603	219	800	**450**
Phosphorus (mg)	781	183	808	172	620	250
Iron (mg)	11	33	7	2	8	**7**
Copper (mg)	0.79	0.91	0.66	0.22	0.4	1
Zinc (mg)	5	2	6	2	5	**4.3**
Magnesium (mg)	253	74	244	53	150	230
Chromium (µg)	43	17	50	23	60	no data
Manganase (mg)	1.1	0.4	1.7	2.1	1.2	0.5
Retinol equivalent. (mg)	0.65	0.83	0.46	0.29	0.4	0.25
D vitamin (µg)	1.4	0.5	1.6	1.3	10	15
α-Tocopherol (mg)	11	4	9	3	6	9
Thiamine (µg)	777	222	749	212	500	**100 µg/MJ**
Riboflavin (µg)	1071	348	1052	316	800	**600**
Vitamin B_6_- Pyridoxine (µg)	1284	829	1239	386	500	**600**
Cobalamin (µg)	2.4	1.6	2.5	1.6	0.7	1.5
Vitamin C (mg)	104	66	53	32	50	**20**
Niacin equivalent (mg)	18	5	9	3	9	**1.6 (mg NE/MJ)**
Folate (µg DFE)	122	49	102	44	100	**120**
Pantothenic acid (mg)	3	1	3	1	2	4

* PRIs are presented **in bold type** and AIs in ordinary type.

**Table 2:** Dietary energy and nutrient intakes of healthy, 4- to 6-year-old pre-school children in Hungary.

Daily Energy/Nutrient Intake	Survey in 2013 4–6 Years (*n* = 135) Mean Age: 5.27 Years Min Age: 4 Years Max Age: 6.62 Years		Survey in 2016 4–6 Years (*n* = 408) Mean Age: 5.3 Years Min Age: 4 Years Max Age: 6.9 Years		Hungarian Recommendation 4–6 Years	EFSA Recommandation * 4–6 Years
	Mean	SD	Mean	SD		
Energy (kcal)	**1503**	270	1689	305	1700	5.4 MJ/day day at PAL = 1.4
Protein (g)	55	11	63	12	54	0.85 g/kg bw/d
Protein (E%)	16	7	16	3	13	-
Fat (g)	49	10	63	15	55	-
Fat (E%)	30	5	35	8	30	20–35
Carbohydrate (g)	207	49	215	43	236	154–206
Carbohydrate (E%)	55	6	52	11	57	45–60
Cholesterol (mg)	192	79	232	75	170	no data
Dietary fiber (g)	14	5	18	10	19	14
Water (mL)	1092	298	1037	508	1600	1600
Sugar (E%)	12	5	10	4	10	10
Sodium (mg)	2497	730	3469	852	700	500
Potassium (mg)	1997	567	2096	505	1400	1100
Calcium (mg)	620	215	635	225	800	**800**
Phosphorus (mg)	783	177	855	179	620	440
Iron (mg)	14	39	8	2	8	**7**
Copper (mg)	0.7	0.5	0.7	0.3	0.6	1
Zinc (mg)	6	1	6	2	6	**5.5**
Magnesium (mg)	253	61	262	59	200	230
Chromium (µg)	46	22	53	22	80	no data
Manganase (mg)	1.1	0.4	1.7	1.2	1.7	1
Retinol equivalent (mg)	0.5	0.4	0.5	0.4	0.5	0.3
D vitamin (µg)	1.4	0.9	1.5	0.7	10	15
α-Tocopherol (mg)	17	46	9	3	7	9
Thiamine (µg)	816	259	781	220	700	**100 µg/MJ**
Riboflavin (µg)	1077	350	1104	339	1000	**700**
Vitamin B_6_- Pyridoxine (µg)	1210	376	1349	441	600	**700**
Cobalamin (µg)	3.2	6.2	2.6	1.9	1	1.5
Vitamin C (mg)	87	67	56	34	50	**30**
Niacin equivalent (mg)	19	6	22	5	11	**1.6 (mg NE/MJ)**
Folate (µg DFE)	114	54	105	40	130	**140**
Pantothenic acid (mg)	3	2	3	1	3	4

* PRIs are presented **in bold type** and AIs in ordinary type.

**Table 3:** Percentage of children with excess, adequate or insufficient nutrient intake I.

Daily Energy/Nutrient Intake	Survey in 2013 2.5–4 Years (*n* = 51) Mean Age: 3.3 Years Min Age: 2.6 Years Max Age: 3.9 Years			Survey in 2016 2.5–4 Years (*n* = 148) Mean Age: 3.4 Years Min Age: 2.9 Years Max Age: 3.9 Years		
	Low	Normal	High	Low	Normal	High
Energy	0	100	0	1	72	27
Protein	0	61	39	0	46	54
Fat	6	63	31	1	45	54
Carbohydrate	0	94	6	1	88	11
Cholesterol	8	47	45	1	30	69
Dietary fiber	25	62	14	9	75	16
Daily fluid intake	22	75	4	44	54	2
Sugar	20	53	27	9	53	39
Sodium	0	0	100	0	0	100
Potassium	0	8	92	0	7	93
Calcium	39	57	4	46	49	5
Phosphorus	0	59	41	1	53	46
Iron	25	73	2	16	78	6
Copper	0	18	82	1	22	76
Zinc	12	73	16	5	67	28
Magnesium	2	18	80	0	18	82
Chromium	55	43	2	49	35	16
Manganase	24	65	12	14	51	35
Retinol equivalent	22	33	45	21	56	23
Vitamin D	100	0	0	99	1	1
Tocopherol	4	18	78	1	41	58
Thiamine	2	27	71	1	36	64
Riboflavin	2	53	45	5	48	47
Pyridoxine	0	0	100	0	5	95
Cobalamin	0	6	94	0	4	96
Vitamin C	8	24	69	32	39	28
Niacin equivalent	0	8	92	0	3	97
Folate	12	55	33	20	38	43
Pantothenic acid	2	45	53	5	50	45

Low < 70% reference value; Adequate: 70–130% reference value; High: >130% reference value.

**Table 4:** Percentage of children with excess, adequate or insufficient nutrient intake II.

Daily Energy/Nutrient Intake	Survey in 2013 4–6 Years (*n* = 135) Mean Age: 5.27 Years Min Age: 4 Years Max Age: 6.62 Years			Survey in 2016 4–6 Years (*n* = 408) Mean Age: 5.3 Years Min Age: 4 Years Max Age: 6.9 Years		
	Low	Normal	High	Low	Normal	High
Energy	10	88	2	3	90	7
Protein	4	89	7	0	74	26
Fat	14	84	2	3	73	25
Carbohydrate	20	75	5	12	86	2
Cholesterol	16	58	27	3	50	47
Dietary fiber	50	48	2	22	73	5
Daily fluid intake	59	39	1	68	31	1
Sugar	11	49	40	18	59	23
Sodium	0	0	100	0	0	100
Potassium	1	40	59	0	31	69
Calcium	43	54	3	39	55	6
Phosphorus	1	59	41	0	42	58
Iron	21	71	8	8	81	11
Copper	4	71	25	3	68	29
Zinc	16	75	9	10	76	14
Magnesium	1	59	41	0	51	48
Chromium	70	27	2	60	36	4
Manganase	65	34	1	29	58	13
Retinol equivalent	36	42	22	32	53	16
Vitamin D	100	0	0	100	0	0
Tocopherol	3	41	56	4	46	50
Thiamine	11	55	34	7	68	25
Riboflavin	7	75	19	9	65	26
Pyridoxine	1	7	92	0	6	94
Cobalamin	1	12	87	1	10	89
Vitamin C	23	30	47	28	41	31
Niacin equivalent	2	16	82	0	4	96
Folate	42	41	17	40	55	5
Pantothenic acid	25	66	9	19	69	12

Low < 70% reference value; Adequate: 70–130% reference value; High: >130% reference value.

## Data Availability

The data presented in this study are available on request from the corresponding authors.
